# Tracing more than two decades of Japanese encephalitis virus circulation in mainland China

**DOI:** 10.1128/jvi.01575-24

**Published:** 2025-02-13

**Authors:** Gairu Li, Xinxin Li, Jie Chen, Phillipe Lemey, Bram Vrancken, Shuo Su, Simon Dellicour, Fabiana Gámbaro

**Affiliations:** 1 Jiangsu Engineering Laboratory of Animal Immunology, Institute of Immunology, College of Veterinary Medicine, Academy for Advanced Interdisciplinary Studies, Nanjing Agricultural University 261674 , Nanjing, China; 2 Sanya Institute of Nanjing Agricultural University 663035 , Sanya, China; 3 Department of Microbiology, Immunology and Transplantation, Rega Institute, KU Leuven 573654 , Leuven, Belgium; 4 Spatial Epidemiology Lab (SpELL), Université Libre de Bruxelles 26659 , Brussels, Belgium; Emory University School of Medicine, Atlanta, Georgia, USA

**Keywords:** Japanese encephalitis virus, genomic epidemiology, phylogeography

## Abstract

Japanese encephalitis is a viral disease caused by the Japanese encephalitis virus (JEV), primarily affecting rural areas of Asia and western Pacific region. China remains one of the main epicenters, experiencing a significant burden of human and animal cases despite vaccination efforts. The ecology of this arbovirus is complex, involving *Culex* mosquitoes as primary vectors, wading birds as natural reservoirs, and pigs as amplifying hosts. Given the virus’s epidemiological importance in China, combined with the country’s expanding pig farming industry and diverse climates, investigating the virus spread and its environmental drivers is needed to address its persistent burden. In this study, we conducted phylogeographic analyses by combining publicly available JEV envelope gene sequences from China and other regions. Our reconstructions revealed multiple introduction events leading to various circulating JEV clades in China, with one predominant clade. Additionally, our analyses showed a diffusion capacity of JEV exceeding previous estimates for co-circulating arboviruses. These differences could be attributed to pig trade or bird migration, calling for further investigations into the drivers of JEV spread.

**IMPORTANCE:**

Japanese encephalitis virus (JEV) is the cause of Japanese encephalitis, a significant health concern in China. Despite being one of the most studied mosquito-borne viruses, no previous studies have combined genomic and geographic data to investigate the spatial epidemiology and dispersal capacity of the virus. In this study, we analyzed genomic, geographic, and environmental data to trace the dispersal history of JEV in China and explore the environmental factors influencing its distribution. Our findings show that JEV circulates predominantly in areas with higher temperatures, dense human and pig populations, and favorable conditions for *Culex* mosquitoes. Notably, our analyses showed a higher diffusion capacity of JEV compared to co-circulating viruses, possibly driven by factors like pig trade and bird migration. Our analysis calls for improved genomic surveillance and establishes a baseline for future studies on the effects of climate change, agricultural practices, and bird migration on JEV circulation.

## INTRODUCTION

Japanese encephalitis virus (JEV), a mosquito-borne orthoflavivirus, is the leading cause of viral encephalitis in many countries across Asia and the western Pacific. The World Health Organization estimates that nearly 68,000 clinical cases of Japanese encephalitis occur globally each year (1). JEV exhibits a complex ecology, maintained in a cycle involving *Culex* mosquitoes and vertebrate hosts, primarily wading birds acting as natural reservoirs and pigs serving as amplifying hosts. Humans become infected through the bite of infected *Culex* mosquitoes, with *Culex tritaeniorhynchus* being the primary vector (1). However, as the JEV viremia in humans is typically too low to infect feeding mosquitoes, humans are considered incidental or dead-end hosts. Other vertebrates such as horses, cattle, goats, dogs, and bats are susceptible to JEV infection, and they, too, act as dead-end hosts. In recent years, evidence for pig-to-pig transmission through oronasal shedding accumulated, further complicating the ecological dynamics of JEV (2, 3).

JEV is distributed throughout East and Southeast Asia, the Indian subcontinent, as well as Australia (1, 4, 5). In China, JEV transmission follows a seasonal pattern with the incidence of Japanese encephalitis cases rising after June, reaching its peak in July and August before starting to decline in September (6, 7). The disease is more prevalent in the southern and central regions of the country (8, 9) where temperature and humidity are higher and allow for rice cultivation (4–6). Indeed, JEV infections occur primarily in rural and agricultural areas, particularly those involved in rice production and flooding irrigation practices (5, 6). Irrigated rice fields serve as breeding sites for *Culex* mosquitoes and can also attract migratory wading birds involved in the transmission cycle. As mosquitoes can easily bite both pigs and humans (3), the close proximity to pig farms further increases the risk of human infections.

In most cases, individuals infected with JEV experience mild symptoms or remain asymptomatic. Less than 1% of individuals will develop more severe symptoms, including encephalitis and, in some cases, neurological complications or death (10). Japanese encephalitis is a vaccine-preventable disease, and China has integrated the JEV vaccine into its national immunization program (11). Thanks to extensive vaccination campaigns, JEV incidence has decreased in China; however, the disease remains a significant burden, particularly in the provinces of Guizhou, Chongqing, Sichuan, and Yunnan, probably due to differences in climatic and socio-economic conditions (9). Concurrently, several countries in Asia, including China, have experienced increases in population growth, pork production, and irrigated rice agriculture, which are all factors that favor JEV spread and increase the risk of human infection (11). Given this context, understanding the JEV circulation dynamics in China and investigating the environmental and anthropogenic factors influencing this dynamic has the potential to help control and prevent future JEV outbreaks.

Previous research has analyzed the spatio-temporal distribution of human JEV cases and the climate factors affecting its distribution (7, 12), as well as conducted phylogenetic analyses addressing the genetic diversity of JEV circulating in China (13–15) or more globally in Asia (16, 17). However, studies placing phylogenetic trees in both temporal and geographic contexts, particularly considering the specific environmental characteristics of the study area, are lacking. Such phylogeographic reconstructions hold significant value as they can offer insights into the patterns and pace of virus circulation, as well as the impact of environment factors on the dispersal dynamics of the virus.

In this study, we applied both discrete and continuous phylogeographic approaches to investigate the introduction into and subsequent spread dynamics of JEV in mainland China and assess the influence of environmental factors on viral spread by analyzing a combined data set of available envelope (E) gene sequences from China and the rest of the world.

## RESULTS

### Introduction events of JEV into China

**Fig 1:**
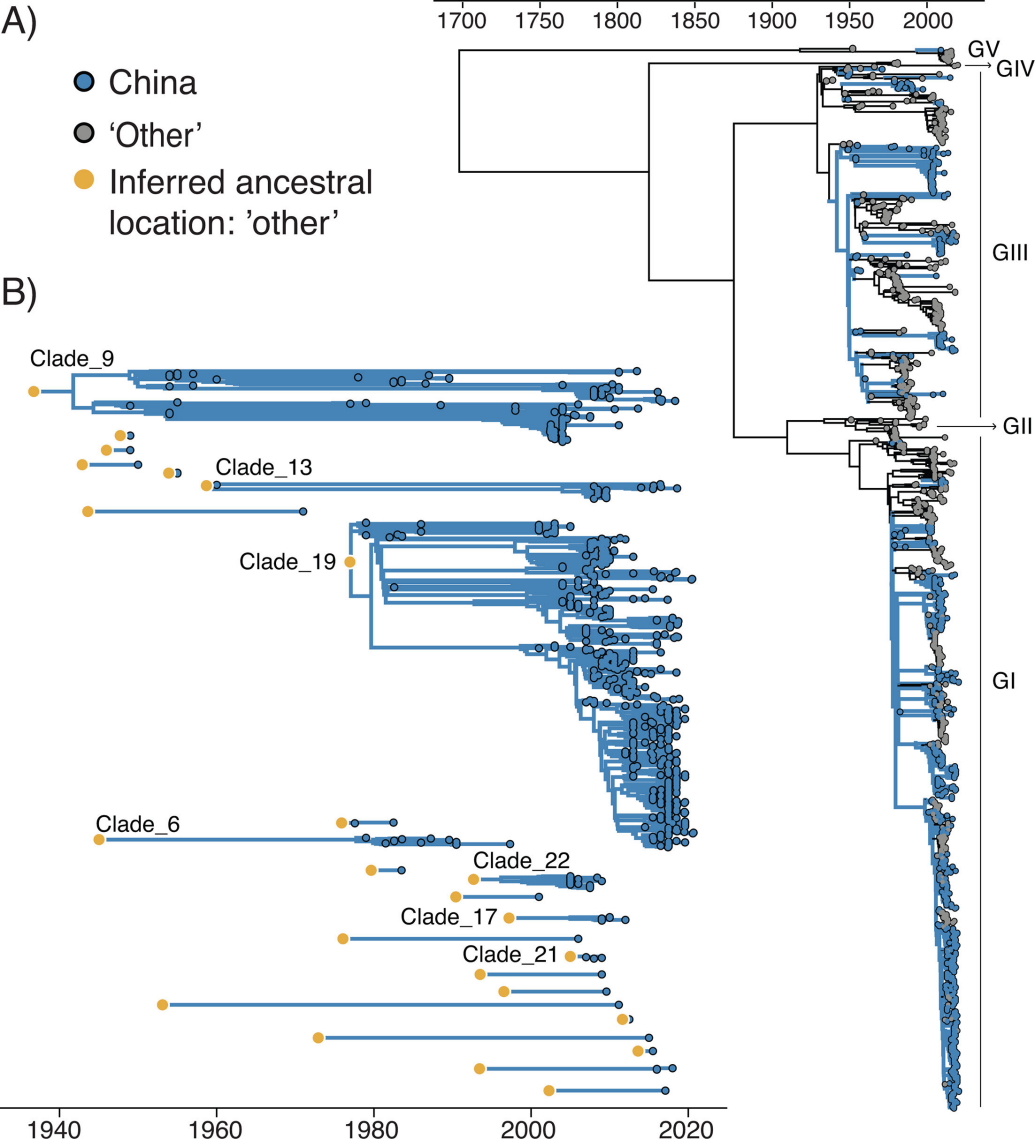
Global JEV phylogeny based on E gene sequences collected until 2020 (*n* = 1,268). (**A**) Maximum clade credibility tree retrieved from a discrete phylogeographic analysis considering two possible locations: “China” and “other,” with branches colored according to the inferred ancestral location. (**B**) Extracted introduction events and associated circulating clades in China from the global JEV phylogeny shown in panel A. A total of 24 distinct introduction events were identified and 7 led to clades with at least three genomic sequences collected in China. Among these clades, only Clade #19 was retained for further analyses, as the others either lacked sufficient temporal signal to calibrate a molecular clock model or contained less than 10 sequences.

To analyze the introduction and dispersal dynamics of JEV in China over the past decades, we combined JEV E gene sequences collected from China with publicly available sequences retrieved from National Center for Biotechnology Information (NCBI) GenBank until 2021, totaling 1,268 sequences. Using this data set, we first generated a maximum-likelihood (ML) phylogeny that placed the JEV sequences collected from China within the JEV genotypes I (GI) and III (GIII), with 100% bootstrap node support (Fig. 1A; Fig. S1).

We then conducted a discrete phylogeographic analysis to delineate clades that corresponded to distinct JEV introductions into mainland China. In this analysis, we considered only two discrete locations: “China” and “other.” From the maximum clade credibility (MCC) tree obtained through this discrete phylogeographic analysis, we identified a total of 24 distinct introduction events. Among these events, seven corresponded to clades with at least three sequences, totaling 631 sampled sequences (Fig. 1B). Notably, Clade #19 encompassed the majority of JEV sequences collected in China (*n* = 469; approximately 72% of the sequences), and represented the largest and oldest clade belonging to the JEV GI. Clades #21 and #22 are nested within Clade #19, indicating potential instances where JEV GI entered the country, exited, and then re-entered. Conversely, Clade #17, which includes six sequences collected between 2009 and 2012, also branches within the GI genotype but falls outside Clade #19, suggesting a separate and more recent JEV introduction into the country (Fig. S1).

### Dispersal dynamics of JEV within China

We subsequently performed a spatially explicit (i.e., continuous) phylogeographic analysis, focusing exclusively on Clade #19 because other clades were of too small size or they lacked sufficient temporal signal (see the Materials and Methods section for further detail). This analysis followed a two-step approach. First, we performed a phylogenetic reconstruction using a skygrid coalescent model as tree prior, setting up yearly time intervals (18). Second, we conducted the continuous phylogeographic analysis averaging over 1,000 post-burn-in trees sampled from the previous step. We incorporated sampling location uncertainty by using the contour of the administrative polygons to delineate a prior range of possible sampling coordinates and estimating the sampling locations within the administrative polygons (19, 20).

**Fig 2:**
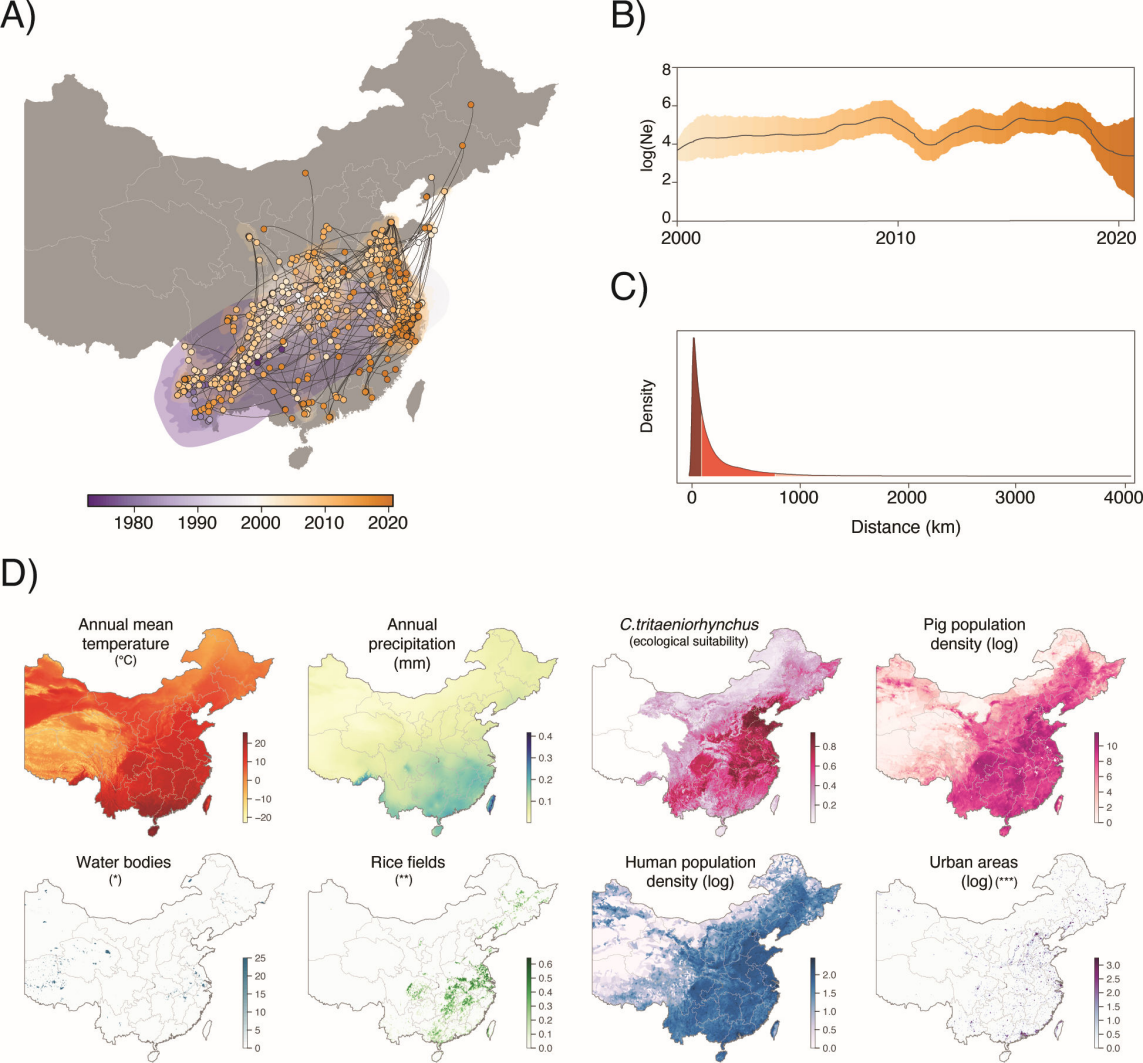
Continuous phylogeographic analysis of JEV in China. (**A**) Continuous phylogeographic reconstruction of the dispersal history of JEV Clade #19. We here map the MCC tree along with 80% high posterior density (HPD) regions reflecting the uncertainty associated with the Bayesian phylogeographic inference. (**B**) Bayesian skygrid plot of the effective population size through time (2000–2020). The posterior mean estimate is indicated by the black line and the colored areas correspond to the 95% HPD. (**C**) Kernel density estimate of geographic distance traveled by phylogenetic branches of 900 posterior trees sampled in the posterior distribution of the continuous phylogeographic inference. Dark red and red-colored areas represent the 50th and 95th percentiles. The 50 and 95% of the branches traveled up to approximately 100 and 800 km, respectively. (**D**) Environmental variables investigated in our landscape phylogeographic analyses. (*) and (***) values represent range of coverage (0 to 25); (**) values represent the proportion of rice cropland per grid cell. Maps were obtained from the Resources and Environmental Sciences Data Platform of the Chinese Academy of Sciences (www.resdc.cn).

While the skygrid coalescent model suggested no global increase of the overall JEV GI population size in the last ~50 years (Fig. 2), we investigated whether the transmission of JEV GI in China followed a seasonal pattern. To explore this, we conducted a second skygrid reconstruction, this time setting up one grid point per month to capture any potential seasonality. Despite most of our samples being collected during the warmer months of June, July, and August (*n* = 423), which correspond to the peak of summer in China, and the geographic distribution of the samples (predominantly from the southern and eastern parts of China as shown by the continuous phylogeographic reconstruction in Fig. S2), we did not infer any discernible seasonal pattern (Fig. 2B). However, it is important to note that our analysis focused on a single clade (Clade #19), the major JEV clade detected in China. Furthermore, the lack of dense sampling from each season might have prevented us from detecting such a pattern.

Our analysis estimated that the date for the most recent ancestor of JEV GI in mainland China to be around 1971 (95% high posterior density [HPD] = [1965–1976]). Although the exact location of the root of the tree could not be precisely inferred, the analysis showed that JEV GI predominantly circulates in the central southeastern part of the country: it initially circulated in the southern provinces of Yunnan, Guangxi, and Guizhou, and then spread to the eastern and northern parts of the country (Fig. S2), with rare very long-distance lineage dispersal events (>1,000 km). Indeed, 50% and 95% of the lineage dispersal events that we inferred occurred within distances of approximately 100 and 800 km, respectively (Fig. 2C). Additionally, we used the continuous phylogeographic reconstructions to estimate the weighted diffusion coefficient (21) of the JEV GI within China to be ~31,000 km^2^/year (95% HPD = [26,874–35,154]).

### Assessing the impact of environmental factors on the dispersal dynamics of JEV lineages

As a mosquito-borne disease, environmental factors can significantly influence Japanese encephalitis circulation. Therefore, we exploited our continuous phylogeographic reconstruction to assess the impact of specific environmental conditions on the dispersal location (22) and velocity (23) of viral lineages. We investigated the impact of several factors including annual mean temperature, annual precipitation, pig population density, mosquito suitability index (i.e., *Culex tritaeniorhynchus*), human population density, and different land cover variables. These land cover variables included urban areas, wetlands, standing water bodies, croplands, irrigated rice fields, total rice fields (both rainfed and irrigated), forest areas, and savannas (Fig. 2D).

By extracting the environmental values at the tree node position, we first explored whether the dispersal location of inferred JEV lineages were associated with specific environmental conditions (22). Our analysis revealed that JEV lineages tended to circulate in areas associated with a relatively higher coverage of water bodies, higher mean annual temperatures and human population density, urban areas, and regions with relatively higher pig population density and mosquito *Culex tritaeniorhynchus* suitability index (Table 1).

**TABLE 1:** Association of environmental factors with the dispersal location of inferred JEV lineages from the main clade circulating in China (here labeled Clade #19)^*a*^

Environmental factor	Tendency of viral lineages to avoid circulating in areas associated with specific environmental conditions	Tendency of viral lineages to preferentially circulate in areas associated with specific environmental conditions
Annual mean temperature	0.0	**32.3**
Annual precipitation	0.1	15.7
Suitability index *C. tritaeniorhynchus*	0.0	**49.0**
Pig population density (log)	0,0	**99.0**
Wetlands	0.2	4.9
Water bodies	0.0	**>99**
Human population density (log)	0.0	**>99**
Urban areas	0.0	**>99**
Total rice	0.1	9.0
Irrigated rice	0.1	8.1
Savannas	0.8	1.2
Forest areas	0.0	**>99**
Croplands	0.3	3.0

^*a*^ We report Bayes factor (BF) support for the association between environmental values extracted at tree node locations, based on 100 trees sampled from the posterior distribution of trees obtained by continuous phylogeographic inference. Following the scale of interpretation of Kass and Raftery (24), we consider a BF value >20 as strong support (in bold).

To evaluate the impact of environmental factors on the dispersal velocity of the virus, we tested the environmental layers as either conductance or resistance factors (25). In short, we used path models (26, 27) to test the association between the dispersal durations and environmental distances associated with phylogenetic branches, with the objective to identify if some of these factors could explain a degree of heterogeneity in lineage dispersal velocity. These analyses indicated that none of the tested environmental factors is significantly associated with heterogeneity in the inferred dispersal velocity of JEV GI lineages. Our results therefore indicate that none of the environmental predictors improves on spatial distance as the best predictor of the dispersal duration of those viral lineages.

## DISCUSSION

In this study, we first conducted a discrete phylogeographic analysis while only considering two sampling locations: China (the study area) and “other” (outside the study area). Given that the aim of this preliminary discrete phylogeographic analysis was to delineate JEV clades circulating in China and corresponding to distinct introduction events into the country, there was indeed no necessity to consider a more detailed set of discrete locations outside the target study area, which would have been associated with an additional computational burden. Moreover, given the notable heterogeneous sampling effort of JEV genomes in the surrounding countries, it would have been anyway challenging to exploit such a discrete phylogeographic reconstruction to precisely infer the origin of the delimited introduction events in China. The strategy of grouping regions/countries outside the study area as “other” to delineate lineage importation events has been applied in several previous studies (28–31). We then conducted a continuous phylogeographic analysis to evaluate the dispersal dynamic of JEV in the country and the environmental factors impacting its distribution.

The discrete phylogeographic analysis identified 24 independent introduction events of JEV into mainland China, with 29% of these (7 out of 24) forming a clade of at least three sequences (Fig. 1B). However, it is important to note that these numbers are likely underestimated due to incomplete or uneven subsampling of JEV in China and neighboring countries. Indeed, in most endemic countries, virus detection predominantly relies on passive surveillance of humans and swine, and to a lesser extent active surveillance efforts targeting mosquitoes (32). The lack of standardized surveillance systems across JEV-endemic countries might lead to differences in sampling and sequencing efforts, both between and within countries, which can significantly impact phylogenetic and phylogeographic reconstructions (33, 34).

Our continuous phylogeographic analysis focused on Clade #19 of GI, representing the largest clade encompassing approximately 72% of JEV sequences sampled in China (Fig. 1). While GIII has historically circulated in China and other countries, GI has recently emerged as the dominant JEV genotype throughout Asia (16). Consistent with previous studies, our reconstruction shows a relatively important circulation of JEV in the southeastern region of China (8, 9, 11).

Additionally, we estimated the weighted diffusion coefficient, a dispersal metric which has been shown to be robust to the sampling size to evaluate and compare the dispersal capacities of various viruses (35). For JEV, we estimated a weighted diffusion coefficient of approximately ~31,000 km^2^/year. This estimate is lower than the diffusion coefficient for West Nile virus, a closely related mosquito-borne virus, in North America (36) (even when not considering the expansion phase of the virus across this continent [35]), but higher than yet similar to—with overlapping 95% HPD intervals—that for the Getah virus in China (37), another mosquito-borne virus also affecting pig population, as well as porcine deltacoronavirus in eastern and southern China (38) (Fig. S2C). These differences in diffusion coefficient estimates could potentially be attributed to variations in ecological dynamics, including differences in host and vector species.

In our study, as shown in Fig. 2C; Fig. S1, the spread of JEV GI in China appears to be mostly characterized by distance events spanning up to 800 km, and with some rare longer events (>1,000 km). While we lack information about the movement patterns of pigs within China to formally test this, considering China is the largest pig producer worldwide (39), it is possible that human-mediated pig trade has contributed to the spread of JEV. Furthermore, most of the JEV infections result in high viremia in pigs, with rare symptomatic disease (11, 40), potentially facilitating the unintentional transport of infected pigs and hampering control strategies. Additionally, unlike for Getah virus or porcine deltacoronavirus, wading birds of the family *Ardeidae*, including herons and egrets, constitute the JEV animal reservoir (4, 5). These birds are widely distributed in the agricultural lands of Central and Southeast China and may travel significant distances from their breeding sites to wintering sites in Southeast Asia, as shown by recent GPS track studies (41, 42). One hypothesis worth investigating is whether bird migration contributes to the relatively high diffusion coefficient estimated for JEV and to the apparently frequent north-to-south dispersal of JEV in China observed in the phylogeographic reconstruction. Future studies that assess the contribution of the role of bird migration in JEV spread would offer valuable insights into the complex ecology of the virus.

Our analyses also suggest that sampled JEV GI lineages have mainly circulated in areas characterized by a higher presence of standing water bodies, higher temperature, human and pig population density, and *Culex* mosquito suitability index, which aligns with current knowledge about JEV ecology. Virus transmission is primarily associated with agricultural areas where rice farming and intensive pig breeding create an optimal environment for mosquito development and transmission of JEV among humans, mosquitoes, and pigs. In contrast, we only found a low association with rice fields (irrigated and “total,” which considers irrigated and rainfed fields) with a Bayes factor support ~9 while these factors are also likely to contribute to the maintenance of the virus circulation. This discrepancy could potentially be due to a limitation inherent to the landscape phylogeographic analysis conducted here: it can be significantly influenced by the spatial heterogeneity of the sampling effort, as the analyzed environmental data are collected at each node of the phylogenetic tree, with half of these positions directly reflecting the sampling process (22). Therefore, while these findings provide valuable insights they should be interpreted with consideration of the methodological limitations.

Recent studies indicate an expansion of the JEV geographic distribution. JEV has been detected in high-altitude areas with detections in human and pig serum samples, as well as in mosquito *Culex tritaeniorhynchus* in Tibet (43, 44). Similarly, human JEV cases have been detected in the province of Xinjiang in northwestern China, historically considered a non-endemic JEV area (45). As seen for other arboviruses, as temperature rises, the mosquito’s habitat potentially expands. Further modeling studies integrating mosquito ecology (e.g., blood-feeding behavior, flying activity), modern pig-rearing facilities, and climate change will be needed to gain a comprehensive understanding of the potential JEV spread in the near future.

While future studies would benefit from standardized surveillance systems to gather more comprehensive data on JEV circulation, our study combines genomic and geographic data to provide insights into the introduction and spread of JEV, particularly JEV GI, in China, as well as the environmental conditions associated with its dispersion. Additionally, the continuous phylogeographic analysis allowed the estimation of the diffusion coefficient, which resulted to be higher than that of another arbovirus—Getah virus—in the same region. Understanding why and how a virus gets established and spreads in a region can be a very complex question, but estimating dispersal statistics, such as a diffusion coefficient, can offer a basis to investigate the invasion and dispersal capacity of a virus.

## MATERIALS AND METHODS

### Collection of viral sequences

We retrieved all publicly available JEV E gene (E) from NCBI GenBank up to 4 June 2021. Only sequences with a minimum length of 1.2 kb and with known collection dates and sampling locations (at least at the province level) were included. Additionally, higher levels of administrative precision, such as city or village information, were obtained from associated publications. In total, 652 geo-referenced sequences were obtained (Table S1). These sequences were combined with JEV E sequences, also retrieved from GenBank, collected around the globe, in particular from Southeast Asia and Australia, totaling 1,268 sequences. Of the 1,268 sequences included in this analysis, 839 were from mosquitoes, 224 from pigs, 164 from humans, 1 from a bird, 15 from other vertebrates, and 24 had no host information available. We here focused on JEV E sequences because it allowed us to incorporate a larger number of sequences into the phylogenetic and phylogeographic analyses. Sequences were aligned using MAFFT (40) and the resulting alignment was manually inspected.

### Preliminary phylogenetic inference

We conducted a preliminary phylogenetic inference through a two-step process. First, we performed a ML phylogenetic inference with IQ-TREE 2.2.0 (46) using default settings and the best-fitting substitution model identified by ModelFinder (47), followed by Ultrafast Bootstrap approximation (48) with 1,000 replicates. Second, the resulting ML tree was time-calibrated using the program TreeTime v0.8.6 (49), with a clock filter value of 8 applied to identify and remove sequence outliers beyond eight interquartile ranges from the regression line.

### Identification of JEV clades introduced into mainland China

To investigate the spread of JEV lineages in mainland China, we conducted a phylogeographic analysis with the discrete diffusion model implemented in BEAST 1.10.4 (50) to identify JEV circulating in the country. To circumvent computational limitations, we used a fixed tree topology obtained from the time-scaled phylogenetic inference obtained with TreeTime in the previous step. The analysis was run for 100 million Markov chain Monte Carlo (MCMC) steps, with samples collected every 1,000 iterations. Convergence and mixing were assessed using Tracer 1.7.2 (51), with all parameters exhibiting effective sample size (ESS) values greater than 200. After discarding the initial 10% of sampled posterior trees as burn-in, an MCC tree was generated with TreeAnnotator 1.10 (52). The resulting MCC tree was used for the identification of distinct JEV clades (i.e transmission chains) introduced into mainland China. This involved comparing the assigned locations of pairs of nodes connected by phylogenetic branches, considering both the most probable location at internal nodes and the sampling location of tip nodes. A JEV clade circulating in China was defined as a clade of at least three viral genomes sampled in China and whose ancestor location was inferred to be outside China.

### Temporal signal assessment

Chinese clades delineated at the previous step were analyzed with TempEst (53) for investigating their associated temporal signal or “clock-likeness.” Given the poor temporal signal of some of the clades or their small size (data not shown), we decided to continue working only with the largest clade (*n* = 469, Clade #19) encompassing approximately 72% of the collected sequences. Additionally, sequences identified as outliers by TempEst were removed from this clade, resulting in a final data set of 461 sequences.

### Time-scaled phylogenetic inference and skygrid reconstruction

Focusing on the main clade, we conducted a time-scaled phylogenetic inference with BEAST 1.10.4 (50) and the Beagle v.4.0 library (54) to enhance computational calculations. For this analysis, we specified a GTR+Γ nucleotide substitution model (55), a relaxed molecular clock to model branch-specific evolutionary rates (45), and the skygrid model (18) as tree prior, setting up one grid point per year. The analysis was run for 1 billion steps, sampling every 100,000 iterations. We assessed convergence and mixing properties of the MCMC using Tracer (51), ensuring that all estimated parameters had associated ESS values greater than 200. Additionally, to investigate JEV seasonality in China, we conducted a second analysis implementing a skygrid model with one grid point per month from 2000 to 2020.

### Continuous phylogeographic reconstruction

To reconstruct the spread of JEV in China, we conducted a continuous phylogeographic analysis focusing on the largest clade (Clade #19). The spatially explicit phylogeographic analysis was performed using the relaxed random walk diffusion model implemented in BEAST 1.10.4 (45), with a Cauchy distribution to model variations among branches. Our continuous phylogeographic analysis was based on 1,000 empirical trees (56) treated as fixed topologies and sampled from the time-scaled phylogenetic inference conducted with BEAST (with a skygrid model set to one grid point per year) as described above. Running a continuous phylogeographic analysis based on empirical trees requires the specification of a specific block in the BEAST XML input file to set a Hamiltonian Monte Carlo operator (see below for the accessibility of our input and output files). Latitude and longitude coordinates were initially assigned to each sequence by randomly drawing a sampling point within the polygon of the administrative area of origin of each sequence. This approach ensures unique sampling coordinates, a requirement for continuous phylogeographic inference. Sampling location uncertainty was then incorporated in our analysis by providing the contour of the administrative polygon of each sequence as a prior range of possible sampling coordinates (20). The MCMC was run for 100 million iterations and sampled every 100,000 iterations. As described in the previous section, we used the program Tracer 1.7.2 to inspect MCMC convergence and mixing properties and verify that estimated parameters had ESS values greater than 200, and we used TreeAnnotator to identify and annotate the MCC tree.

We extracted and visualized the spatio-temporal information embedded in 900 trees sampled from the post-burn-in posterior distribution using the R package “seraphim” (57). We used the “spreadStatistics” function of the same package to estimate the weighted diffusion coefficient. We implemented previously introduced approaches developed by Dellicour et al. to investigate the impact of environmental factors on the dispersal location (22) and dispersal velocity (23) of viral lineages. For a detailed description, refer to the supplemental material.

## Data Availability

R scripts, sequence data, metadata file, and BEAST XML files associated with the phylogeographic analyses are all available at https://github.com/FabiGambaro/JEV_China.

## SUPPLEMENTAL MATERIAL

Supplemental material jvi.01575-24-s0001.docx Supplemental methods, Fig. S1 and S2, and Table S1.

ASM does not own the copyrights to Supplemental Material that may be linked to, or accessed through, an article. The authors have granted ASM a non-exclusive, world-wide license to publish the Supplemental Material files. Please contact the corresponding author directly for reuse.
