# Supplementary material for: Tracing more than two decades of Japanese encephalitis virus circulation in mainland China
Source: J Virol. 2025 Feb 13;99(3):e01575-24. doi: 10.1128/jvi.01575-24 (PMC11915822; doi:10.1128/jvi.01575-24)
Supplement: Supplemental material — Supplemental methods, Fig. S1 and S2, and Table S1. [file jvi.01575-24-s0001.docx]

**Supplementary Materials for:**

**Tracing more than two decades of Japanese encephalitis virus circulation in mainland China**

Gairu Li^1,2^, Xinxin Li^1,2^, Jie Chen^1,2^, Phillipe Lemey^3^, Bram Vrancken^3,4^, Shuo Su^1,2,^†, Simon Dellicour^3,4,^†, Fabiana Gámbaro^4,^†

^1^ Jiangsu Engineering Laboratory of Animal Immunology, Institute of Immunology, College of Veterinary Medicine, Academy for Advanced Interdisciplinary Studies, Nanjing Agricultural University, Nanjing,China

^2^ Sanya Institute of Nanjing Agricultural University, Sanya, China.

^3^ Department of Microbiology, Immunology and Transplantation, Rega Institute, KU Leuven, 3000 Leuven, Belgium

^4^ Spatial Epidemiology Lab (SpELL), Université Libre de Bruxelles, Brussels, Belgium

(†) denotes equal contribution

**This file includes:**

- Methods section
- Figure S1
- Figure S2
- Table S1
- References

**Methods**

*Testing the impact of environmental factors in the dispersal location and velocity of viral lineages*

We implemented a previously introduced method^1,2^ to test the association between different environmental factors (described by raster files) and the dispersal location^3^ and velocity^4^ of JEV GI in China. Both approaches rely on comparing the results from the “as is” inference with results from a null model obtained under a randomisation scheme. These randomised trees were obtained with the “treesRandomisation” function of the “seraphim '' R package. This function uses the inferred trees as a skeleton, maintaining the same time-scaled topology and root location while randomising the geographic position, i.e., latitude and longitude coordinates, for the remaining nodes. These randomisations were constrained within the study area defined by the minimum convex hull encompassing all node positions, while also preventing the occurrence of randomised tree node positions in non-accessible (e.g., sea) areas. Consequently, each pair of inferred and randomised trees differed only in the geographic coordinates associated with their nodes, except for the root node location^1^.

To investigate the impact of environmental factors on the dispersal locations of viral lineages, for each environmental raster, we extracted the environmental values at the tree node positions from a set of 100 inferred and randomised trees. In this way, we obtained a posterior distribution of mean environmental values for each raster for the randomised (*E*_randomised_) and inferred (*E*_inferred_) trees. To determine whether viral lineages tended to circulate or avoid circulating in areas with specific environmental conditions, we estimated the Bayes factor (BF) support by comparing *E*_randomised_ vs *E*_inferred_. Specifically, BF was defined as BF = (*p_e_*/(1-*p_e_*))/(0.5/(1-0.5)). For assessing if the environmental variable (*e*) attracted or repelled viral lineages, *p_e_* was defined as the frequency at which the environmental values from inferred trees were greater or lower than values from randomised trees, respectively.

To assess how environmental factors influenced the dispersal velocity of JEV lineages, we computed an “environmental distance” using the environmental rasters and a null raster whose cell values are all set to 1. We used two different path models — the least-cost^5^ and Circuitscape path models^6^ — to compute these environmental distances. These models can be used to approximate the paths that could have been taken by a phylogenetic branch as it travels from the start to the end location and compute a “weight” to each branch of the tree based on the values of raster cells. In the case of the null raster, where all cells have the same value set to “1”, the weight for each branch is proportional to the geographic distance^1^. Each raster file was tested as a potential resistance or conductance factor, meaning it could either facilitate or impede viral lineage dispersal. We also tested different strengths of conductance and resistance by transforming the original raster cell values with a rescaling parameter *k* (*k* = 10, 100, and 1000), using the formula *v*_t_ = 1+*k*(*v*_ß_/*v*_max_) where v_t_ and *v*_ß_ corresponds to the transformed and original cell values, respectively and *v*_max_ the maximum cell value recorded in the raster. Using different values of *k* allows testing different values of resistance or conductance relative to the null raster where *k* = 1. For each resulting environmental raster (*n* = 54, considering nine original raster files, three different *k* values, and testing for both conductance and resistance), we computed the *Q* statistic measuring the correlation between branch durations and environmental distances estimated using a specific environmental raster and the null raster. We obtained a *Q* value from each of the 100 trees sampled from the posterior distribution of the continuous phylogeographic inference, resulting in a posterior distribution of *Q* values. If at least 90% of the *Q* distribution values were positive, *Q* was considered as positive and the environmental factor as a potential explanatory variable. In such cases, the statistical support for the *Q* distribution was compared with the corresponding distribution of *Q* values obtained when computing environmental distances for phylogenetic branches of randomised trees by calculating the Bayes Factor (BF). In our case, none of the environmental factors yielded a *Q* distribution with at least 90% positive values.


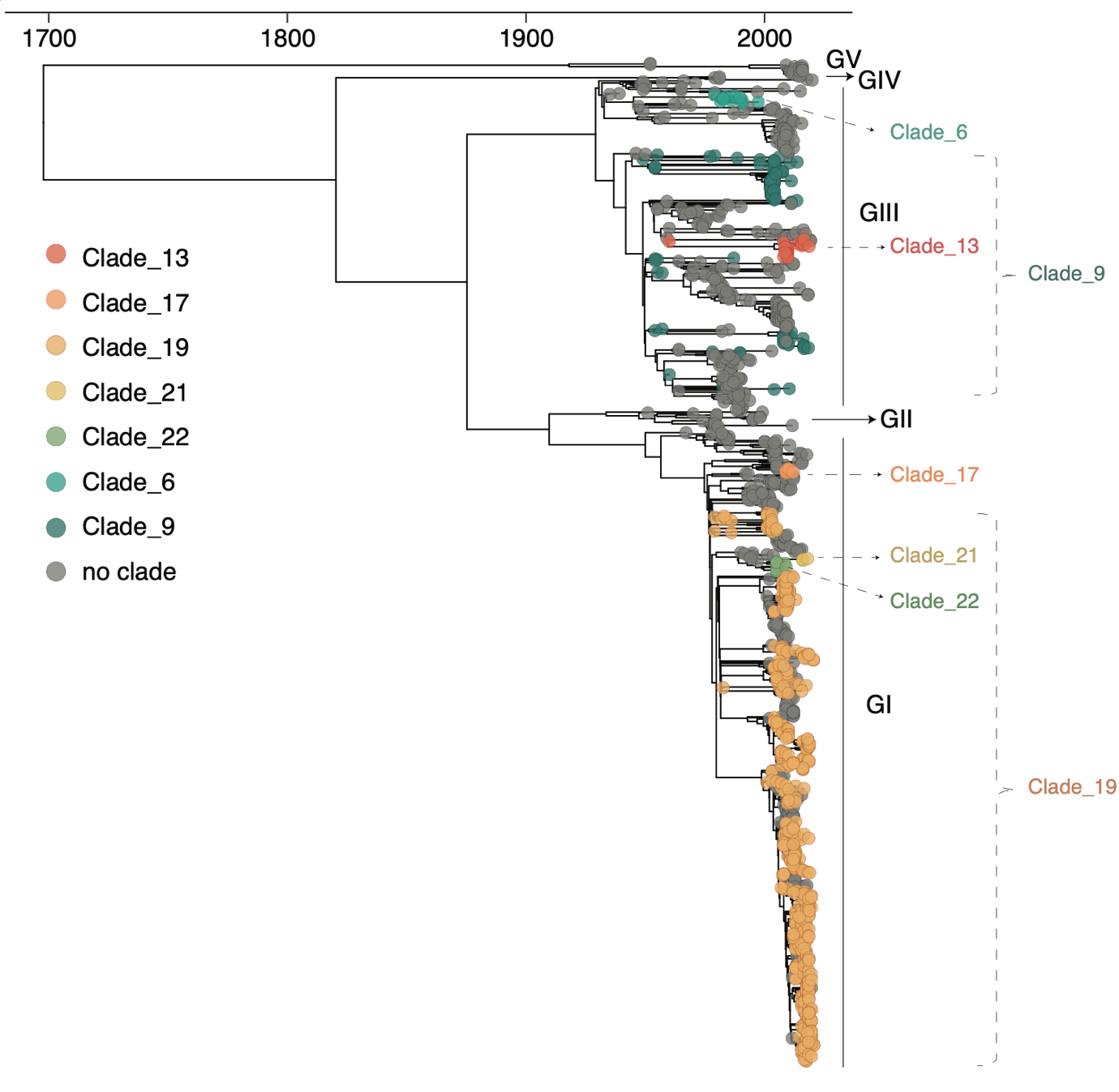


**Figure S1:** **global JEV phylogeny based on E gene sequences collected until 2020 (*n* = 1,268).** The figure displays the maximum clade credibility (MCC) tree obtained from the discrete phylogeographic analysis. Tip nodes of the trees are coloured according to the seven main circulating clades corresponding to distinct introduction events into China, and we also indicate the genotypes (G) corresponding to the different clades of the tree.


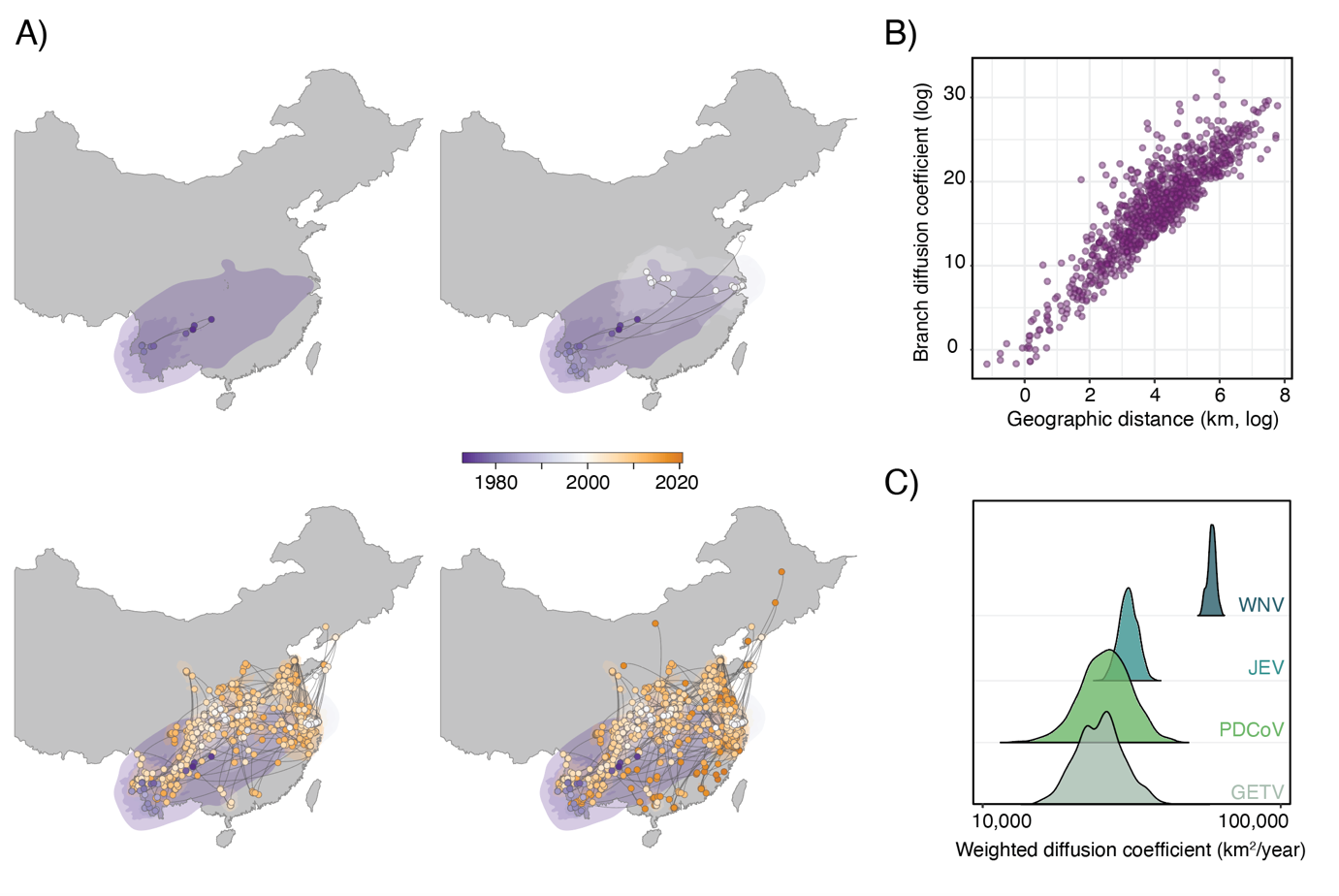


**Figure S2: continuous phylogeographic reconstruction of JEV genotype I (GI) in China.** A) Snapshots of the spatio-temporal spread of JEV GI in China as inferred by a continuous phylogeographic analysis. The four snapshots were taken in 1980, 2000, 2010, and 2020, respectively. We here display the maximum clade credibility (MCC) together with the 80% high posterior density (HPD) regions, indicating the uncertainty associated with the Bayesian phylogeographic inference. Internal and tip nodes were coloured according to the inferred time of occurrence and sampling time, respectively. B) Scatterplot displaying the diffusion coefficient estimated for each branch of the maximum clade credibility (MCC) tree retrieved from the continuous phylogeographic inference (log-transformed values) against the great-circle geographic distances (log-transformed values) travelled by those phylogenetic branches. C) Posterior distribution of the weighted diffusion coefficient (km^2^/year) metric estimated through continuous phylogeographic inference for JEV Clade #19 and for the following viruses: West Nile virus (WNV) in North America^2^, Porcine deltacoronavirus (PDCoV) in China^7^, and Getah virus (GETV) in China^8^. Maps were obtained from the Resources and Environmental Sciences Data Platform of the Chinese Academy of Sciences (http://www.resdc.cn).

**Table S1. Global JEV E-gene sequences (n=1268) and associated metadata used in this study.**

<https://github.com/FabiGambaro/JEV_China/blob/main/supplementary_info/Table_S1.xlsx>

**References**

1. Dellicour, S., Rose, R. & Pybus, O. G. Explaining the geographic spread of emerging epidemics: a framework for comparing viral phylogenies and environmental landscape data. *BMC Bioinformatics* **17**, 82 (2016).

2. Dellicour, S. *et al.* Epidemiological hypothesis testing using a phylogeographic and phylodynamic framework. *Nat Commun* **11**, 5620 (2020).

3. Dellicour, S. *et al.* Using phylogeographic approaches to analyse the dispersal history, velocity and direction of viral lineages — Application to rabies virus spread in Iran. *Molecular Ecology* **28**, 4335–4350 (2019).

4. Dellicour, S. *et al.* Using Viral Gene Sequences to Compare and Explain the Heterogeneous Spatial Dynamics of Virus Epidemics. *Mol Biol Evol* **34**, 2563–2571 (2017).

5. Dijkstra, E. W. *A Note on Two Problems in Connexion with Graphs. Numeriske Mathematik, 1, 269–271*. (1959).

6. McRae, B. H. Isolation by resistance. *Evolution* **60**, 1551–1561 (2006).

7. He, W.-T. *et al.* Genomic Epidemiology, Evolution, and Transmission Dynamics of Porcine Deltacoronavirus. *Mol Biol Evol* **37**, 2641–2654 (2020).

8. Zhao, J. *et al.* Early Genomic Surveillance and Phylogeographic Analysis of Getah Virus, a Reemerging Arbovirus, in Livestock in China. *Journal of Virology* **97**, e01091-22 (2022).
